# Breast Mass Revealing Hidden Lung Cancer

**DOI:** 10.7759/cureus.77429

**Published:** 2025-01-14

**Authors:** Betsalel Adout, Imad Karam, Kafi Thomas, Sayed Ali, Aye M Thida, Sayra Ilyas, Rachelle Hamadi, Shoushtari Alavi, Maksim Agaronov, Edwin Chiu

**Affiliations:** 1 Hematology and Medical Oncology, State University of New York (SUNY) Downstate Medical Center, Brooklyn, USA; 2 Pathology and Laboratory Medicine, State University of New York (SUNY) Downstate Medical Center, Brooklyn, USA; 3 Pathology and Laboratory Medicine, Kings County Hospital, Brooklyn, USA

**Keywords:** lung adenocarinoma, metastasis, phylogenetics, prognosis, rare breast mass

## Abstract

Lung cancer is the second most common cancer and the leading cause of cancer-related deaths in the United States, frequently metastasizing to the brain, bones, liver, and adrenal glands. However, it is uncommon for lung malignancies to metastasize to the breast, and is generally assumed to carry a poor prognosis.

We present the case of a 62-year-old female who presented for an annual physical exam and complained of persistent neck pain without neurological deficit. A screening mammogram revealed a 0.4 cm right breast mass. A biopsy of the mass showed carcinoma with micropapillary features. Immunohistochemistry was positive for cytokeratin (CK) 7 and thyroid transcription factor 1 (TTF-1)and negative for estrogen receptor (ER), progesterone receptor (PR), and human epidermal growth factor receptor 2 (HER2), indicating a primary lung origin. Next-generation sequencing (NGS) was negative for a targetable mutation. The patient was treated for metastatic lung adenocarcinoma with carboplatin, pemetrexed, and pembrolizumab for 4 cycles followed by maintenance with pemetrexed and pembrolizumab. Per response evaluation criteria in solid tumors (RECIST) the patient continues to have stable disease.

Imaging and immunohistochemistry (IHC) are critical in differentiating between primary and metastatic lesions. Additionally, using phylogenetic analysis to understand tumor evolution allows for valuable insights into how metastases develop and spread, thus assisting in personalized treatment strategies. Early and accurate diagnosis is essential for appropriate treatment planning leading to improved patient outcomes.

## Introduction

In the United States, lung cancer is the second most prevalent cancer in both men and women, following breast cancer in women and prostate cancer in men. It is the leading cause of cancer-related deaths, accounting for approximately 20% of all cancer deaths in the United States [1]. While lung cancer frequently metastasizes to the brain, bones, liver, and adrenal glands, metastasis to the breast is rare, occurring in only 0.5%-3% of cases [2]. We present a case of a 62-year-old woman with metastatic lung adenocarcinoma involving the breast, spine, and adrenal glands. 

## Case presentation

A 62-year-old woman presented for her annual physical exam with complaints of persistent neck and upper back pain without neurological deficits. Prior computed tomography (CT) imaging from her home country revealed a lytic lesion in the C5 vertebra. A subsequent magnetic resonance imaging (MRI) scan showed a pathologic compression fracture extending from C4-C5 to C5-C6. 

**Figure 1:**
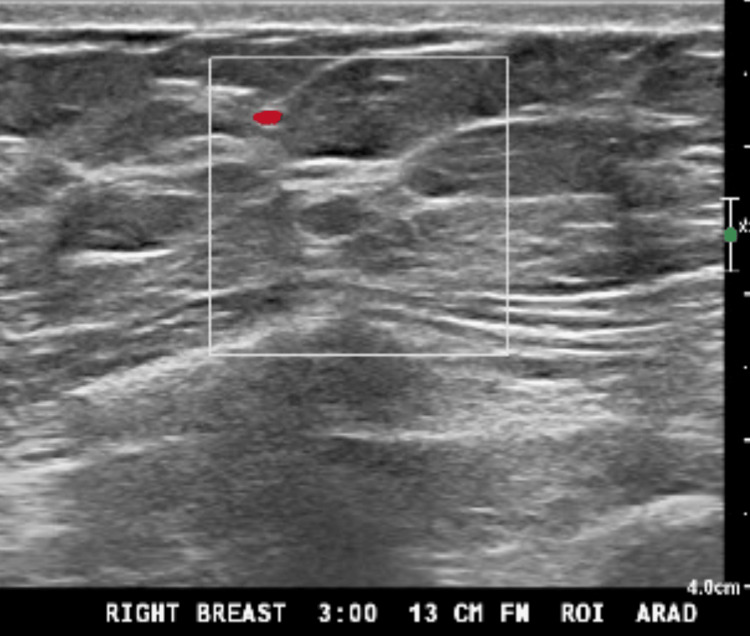
Ultrasound of the right breast, 13 cm from the nipple, with 0.4 cm mass

**Figure 2:**
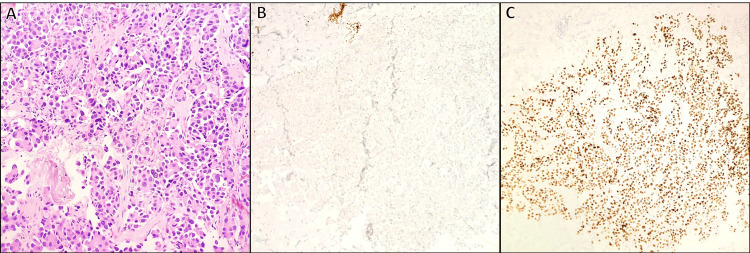
Breast biopsy showing tufts of epithelial cells without fibrovascular cores (micropapillae) with mildly pleomorphic nuclei and prominent nucleoli (A, H&E 200x). Immunohistochemical staining revealed that the malignant cells are negative for breast markers, including GATA-3 (B, H&E 100x), ER, and TRPS1, while positive for TTF-1 (C, H&E 100x) and CK7, highly suggestive of the pulmonary origin of the tumor

**Figure 3:**
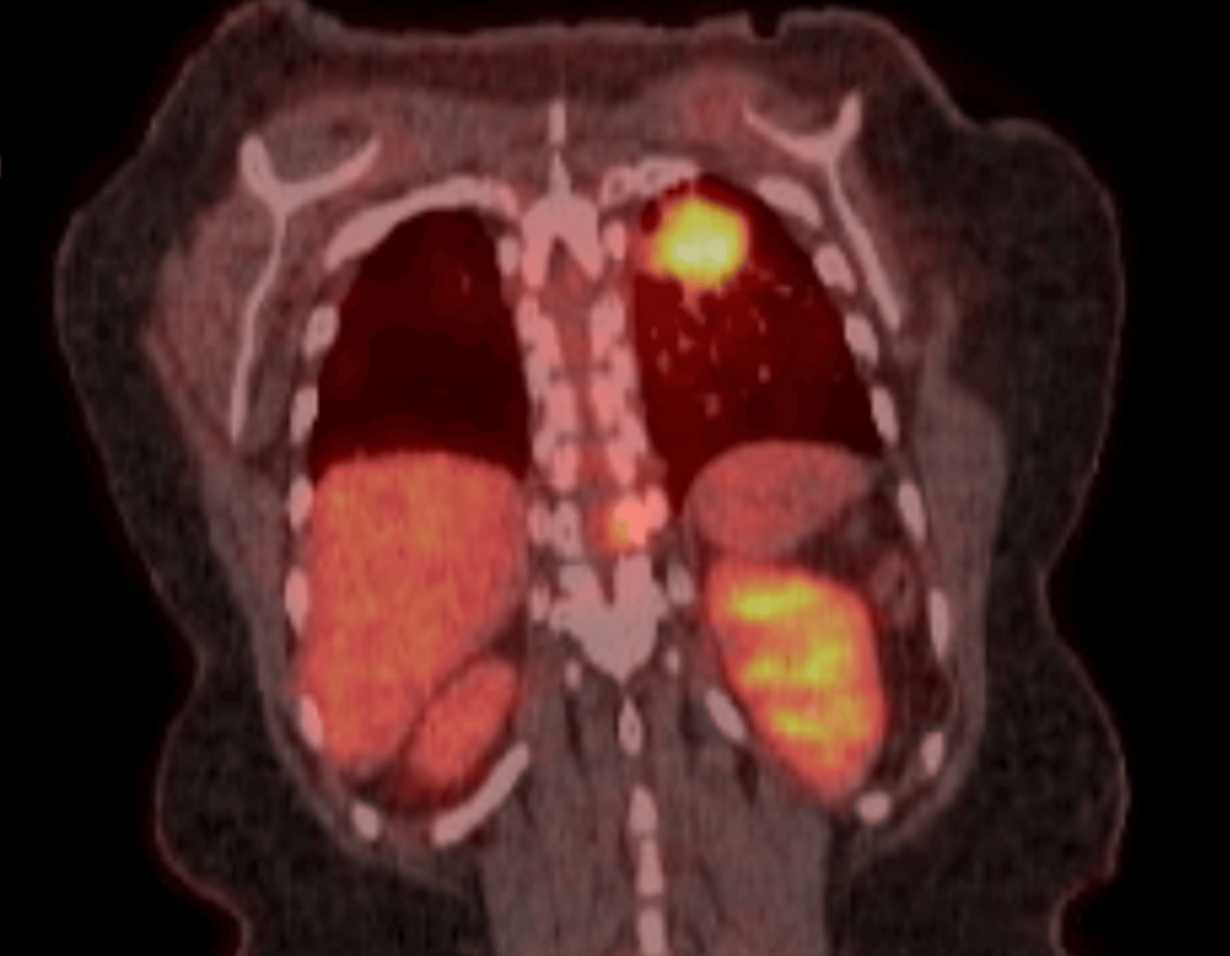
Positron emission tomography-computed tomography scan showing 2.9 x 2.4 x 2.8 cm FDG-avid right upper lung mass (SUV max 5.6)

She also had a right breast mass in the lower inner posterior quadrant, detected on a screening mammogram. A follow-up diagnostic mammogram with tomosynthesis revealed a 0.4 cm lesion (Figure 1). The lesion was classified as Breast Imaging Reporting & Data System (BIRADS) 4, highly suspicious for malignancy. An ultrasound (US)-guided core needle biopsy confirmed adenocarcinoma with micropapillary features. Immunohistochemical analysis was positive for cytokeratin (CK) 7 and thyroid transcription factor 1 (TTF-1) and negative for GATA-3, estrogen receptor (ER), progesterone receptor (PR), human epidermal growth receptor (HER) 2, paired-box gene (Pax) 8, and tumor protein 63 (p63) (Figure 2). These findings suggested a primary lung adenocarcinoma rather than a primary breast carcinoma. Positron emission tomography-computed tomography (PET-CT) revealed a 2.9 x 2.4 x 2.8 cm fluorodeoxyglucose (FDG)-avid right upper lung mass (maximum standardized uptake value (SUVmax) 5.6) (Figure 3), multiple pulmonary nodules, and extensive osseous lesions throughout the axial and appendicular skeleton (SUVmax 4.9). It also showed an FDG-avid destructive lytic lesion at C5 with epidural extension and FDG-avid lesions in bilateral adrenal glands (SUVmax 2.7). Imaging further supported the lung was the primary origin of the malignancy. 

The patient received palliative radiation therapy to the C4-T1 spine and was diagnosed with stage IV lung adenocarcinoma metastatic to the breast, bones, and possibly to the adrenal glands. Next-generation sequencing (NGS) of the tumor was negative for oncogenic driver mutations. Programmed cell death ligand (PD-L) 1 tissue polypeptide-specific antigen (TPS) was 2%. She began the standard treatment regimen: four cycles of carboplatin, pemetrexed, and pembrolizumab, followed by pembrolizumab and pemetrexed maintenance therapy. Her most recent follow-up CT scan shows stable disease with no new lesions. 

## Discussion

In the United States, lung cancer is the second most prevalent cancer across all genders, following breast cancer in women and prostate cancer in men. It is the leading cause of cancer-related deaths, accounting for approximately 20% of all cancer deaths in the United States [1]. Primary lung cancers most frequently metastasize to the nervous system, bones, liver, respiratory system, and adrenal glands [3]. 

Although nearly all malignancies could theoretically metastasize to the breast, the rate of metastasis from extra-mammary sites, such as in the case presented here, is only 0.5%-3% [2]. This rarity may be explained by the breast's poor blood supply and its large areas of fibrous tissue, which provide resistance to metastasis [4]. Metastatic lung cancer is classified as stage IV, with a five-year survival rate of only 5%-7% and a median survival time of less than one year [5]. Breast metastases from non-breast malignancies are typically associated with a poor prognosis [6]. While metastases to the breast can originate from malignant melanoma, lymphoma, lung cancer, ovarian carcinoma, soft tissue sarcoma, and gastrointestinal and genitourinary tumors, they most often arise from contralateral breast carcinoma [7]. Although there are no definitive data on these specific metastases, a review of multiple case reports suggests a consistently poor prognosis (Table 1).

**Table 1:** Case reports N/A: not available.

Author	Age, sex	Chief complaint	Primary tumor location	Outcome
Ji et al. [8]	Case 1: 49, female Case 2: 40, female	Painless mass in the left breast (three months) and right breast mass	Lung adenocarcinoma (both)	Case 1 refused treatment and died five months after diagnosis Case 2 died eight months after the treatment
Fulciniti et al. [9]	59, female	Poorly delimited mass, upper inner quadrant, skin dimpling	Not available	Still alive 14 months after the diagnosis
Klingen et al. [10]	79, female	Subareolar tumor mass, left breast	Not available	Died one month after the diagnosis of the metastasis
Maounis et al. [11]	73, female	Painless mass, skin redness, upper outer quadrant, left breast	Lung cancer unspecified	Died six months after the diagnosis of the metastasis
Sousaris et al. [12]	55, female	Lung adenocarcinoma diagnosis, follow-up CT	Lung adenocarcinoma	N/A
Wang et al. [13]	Case 1: 40, unspecified Case 2: 49, female	Right breast mass in both patients	Spindle cell neuroendocrine carcinoma; small-cell lung carcinoma	Case 1 died seven months after the treatment Case 2 refused chemo and died after eight months
Malek et al. [14]	80, female	Erythema, axillary lymphadenopathy, left breast	Lung adenocarcinoma	N/A
Zhu et al. [15]	36-76 years old (17 female and five male patients)	N/A	Eleven adenocarcinomas, seven small-cell, four squamous	Eleven patients' data N/A; others died within one year, median survival 4.0 months (0.5-11.8)
Wang et al. [16]	49-70 years old (six female and one male patients)	Palpable lesion (six cases), edema (four), pain (one), asymptomatic (one)	Five adenocarcinomas, one large-cell, one small-cell	N/A
Cao et al. [17]	Early 50s, female	Painless mass in the right breast	Lung cancer unspecified	Lost to follow-up three years after the diagnosis

Distinguishing metastatic malignancy from primary breast carcinoma can be challenging, particularly when there are no new or concerning respiratory symptoms, as in the case of primary lung malignancy reported here. Breast metastases typically occur in the subcutaneous layer and are often palpable. Primary breast cancers commonly arise from deeper glandular epithelium and may also be palpable if sufficiently large [8]. Metastatic breast tumors may appear similar to primary breast cancer on mammography, often presenting as multifocal lesions, typically bilateral, with a round shape and well-defined margins, and rarely displaying microcalcifications [18]. 

Pathologic diagnosis of metastatic cancers to the breast, even with a known primary tumor, can be difficult due to histologic similarities to primary breast carcinoma [9]. As such, immunohistochemical studies are necessary to distinguish primary breast cancers from other primary malignancies metastasizing to the breast. In this case, TTF-1 and CK7 positivity is characteristic of lung adenocarcinoma and less likely to be associated with primary breast cancer [19]. 

Phylogenetic tools are used to understand tumor spread by identifying the sequence of driver mutations involved in the metastatic process. By identifying the sequence of driver mutations in the metastatic process, these methods leverage genomic data to trace how tumors disseminate [20]. Tumors may exhibit a star topology, where all metastases stem directly from the primary tumor, or, more commonly, a more complex tree topology, where metastases evolve from one another [21]. While these phylogenetic methods enhance our understanding of tumor evolution, their application in personalized treatment strategies remains under investigation. 

Despite the difficulties in differentiating between a primary lung tumor metastasizing to the breast and a primary breast tumor, accurate diagnosis is crucial for appropriate therapeutic management. Alternative primary malignancies should be considered when patients present with one or more breast masses lacking in situ components, triple-negative but not high-grade features, or poorly differentiated characteristics with an aggressive clinical course unresponsive to treatment [22].

## Conclusions

Lung cancer infrequently metastasizes to the breast. We present here one of these rare occurrences. This case emphasizes the importance of a comprehensive diagnostic workup, including imaging, immunohistochemical analysis, and NGS. Although metastatic lung cancer to the breast generally carries a poor prognosis, our case demonstrated a favorable response to therapy, stressing the critical role of accurate diagnosis and tailored treatment in managing advanced disease. Phylogenetics tools can be used to understand tumor spread based on genomic data sets, but their application in clinical settings remains to be determined.
